# Pre-HAART CD4+ T-lymphocytes as biomarkers of post-HAART immune recovery in HIV-infected children with or without TB co-infection

**DOI:** 10.1186/s12879-020-05458-w

**Published:** 2020-10-15

**Authors:** Vivek Gopalakrishnan, Eliezer Bose, Usha Nair, Yuwei Cheng, Musie Ghebremichael

**Affiliations:** 1 Johns Hopkins University Department of Biomedical Engineering, 3510 N Charles Street, Baltimore, MD 21218 USA; 2 School of Nursing at MGH Institute of Health Professions, 36 1st Ave, Charlestown, MA 02129 USA; 3 Ragon Institute and Harvard Medical School, 400 Tech Square, Cambridge, MA 02129 USA; 4 College of the Holy Cross, 1 College St, Worcester, MA 01610 USA; 5 Ragon Institute of Harvard, MGH and MIT, 400 Technology Square, Cambridge, MA 02129 USA

**Keywords:** HIV, TB, HAART, ROC curves, CD4+ T-lymphocytes; immune recovery

## Abstract

**Background:**

Infection with the Human Immunodeficiency Virus (HIV) dramatically increases the risk of developing active tuberculosis (TB). Several studies have indicated that co-infection with TB increases the risk of HIV progression and death. Sub-Saharan Africa bears the brunt of these dual epidemics, with about 2.4 million HIV-infected people living with TB. The main objective of our study was to assess whether the pre-HAART CD4+ T-lymphocyte counts and percentages could serve as biomarkers for post-HAART treatment immune-recovery in HIV-positive children with and without TB co-infection.

**Methods:**

The data analyzed in this retrospective study were collected from a cohort of 305 HIV-infected children being treated with HAART. A Lehmann family of ROC curves were used to assess the diagnostic performance of pre- HAART treatment CD4+ T-lymphocyte count and percentage as biomarkers for post-HAART immune recovery. The Kaplan–Meier estimator was used to compare differences in post-HAART recovery times between patients with and without TB co-infection.

**Results:**

We found that the diagnostic performance of both pre-HARRT treatment CD4+ T-lymphocyte count and percentage was comparable and achieved accuracies as high as 74%. Furthermore, the predictive capability of pre-HAART CD4+ T-lymphocyte count and percentage were slightly better in TB-negative patients. Our analyses also indicate that TB-negative patients have a shorter recovery time compared to the TB-positive patients.

**Conclusions:**

Pre-HAART CD4+ T-lymphocyte count and percentage are stronger predictors of immune recovery in TB-negative pediatric patients, suggesting that TB co-infection complicates the treatment of HIV in this cohort. These findings suggest that the detection and treatment of TB is essential for the effectiveness of HAART in HIV-infected pediatric patients.

## Background

Of the 38 million people worldwide living with Human Immunodeficiency Virus (HIV), it is estimated that 2.8 million are children (< 19 years) [1]. According to the standard of care for clinical and laboratory monitoring of pediatric HIV infection, markers such as plasma HIV RNA and CD4+ T-lymphocyte count should be assessed routinely [2]. However, the majority of HIV-infected children live in sub-Saharan Africa [3], where such routine monitoring is often unavailable [4, 5]. Due to prohibitive equipment and reagent costs, as well as a lack of laboratory facilities and trained personnel, clinics in resource-limited settings are often ill-equipped to measure these markers as regularly as recommended [6]. Furthermore, even in places where facilities are available, most patients cannot access them consistently [6]. The absence of such longitudinal marker measurements makes it difficult to monitor HIV-infected children on highly active antiretroviral therapy (HAART) regimens, which typically last multiple years. Therefore, methods enabling the prediction of post-HAART outcomes from existing patient data are of vital necessity as they provide a cost-effective alternative to routine monitoring in resource-limited settings. Predictive models of HIV disease prognosis will be more clinically useful if they account for the effect opportunistic co-infections, such as tuberculosis (TB) [7].

HIV and TB co-infection, in particular, poses a significant global health challenge as one in three HIV-positive individuals is estimated also to be infected with TB [8]. Sub-Saharan Africa bears the brunt of these dual epidemics, accounting for 79% of all TB co-infected patients worldwide [8, 9]. Furthermore, the two pathogens act synergistically to worsen patient outcomes: TB is the most common opportunistic disease and leading cause of death amongst HIV-infected individuals; correspondingly, areas with the highest prevalence of HIV infections have seen the greatest increase in the incidence of TB over the past 20 years [10, 11]. The nature of these diseases to potentiate one another changes the approaches one must take when attempting to treat both infections concurrently.

Previous medical research has highlighted many challenges in treating HIV in the presence of TB co-infection [12]. For instance, drug-to-drug interactions between rifampin, one of the most commonly used TB antibiotics worldwide, and various HIV highly active antiretroviral therapies (HAARTs) can yield unintended therapeutic consequences [13]. While rifampin targets TB by inhibiting bacterial RNA polymerase, the drug also induces cytochrome P450 (CYP), a hemoprotein critical for the metabolism of drugs and other foreign molecules [14, 15]. This can accelerate the degradation of HIV-targeting protease inhibitors, resulting in subtherapeutic concentrations of HAARTs in TB co-infected patients [16]. Furthermore, the existence of multiple drug-resistant (MDR) TB strains [17], varied TB presentations (including miliary, exudative pleuritis, and tracheobronchial [18]), and differential clinical manifestations in pediatric patients [19] add additional challenges to this problem. Therefore, to account for the inherent complexity of TB co-infection, models of HIV disease prognosis must be designed for specifically defined patient populations.

The majority of findings regarding TB co-infection were derived from studies of HIV-positive adult cohorts; however, investigations on TB co-infection in children are still lacking [20]. Therefore, the main objective of our study was to determine if the impact of pre-HAART CD4+ T-lymphocyte count and percentage on pediatric immune recovery varied based on TB co-infection status. Using data from a cohort of HIV-positive children from Accra, Ghana, we assessed the ability of CD4+ T-lymphocytes to act as biomarkers for immune recovery. Precisely, CD4+ T-lymphocyte counts and percentages measured before the initiation of treatment were used. Using the Receiver Operating Characteristic (ROC) curves, we measured the diagnostic performances of these markers following adjustments for TB co-infection status, the primary covariate of interest. Additional concomitant variables such as age were also accounted for separately.

## Methods

### Study participants

The data analyzed in this retrospective study were collected from a cohort of HIV-infected children treated with highly active antiretroviral therapy (HAART) in Accra, Ghana. The rationale, methods, and recruitment of the participants have been described in detail previously [21]. The cohort consisted of 305 HIV-positive children between 0 and 13 years of age who initiated HAART regimens between June 2004 and December 2009. The study was reviewed and approved by both the Yale University Human Investigation Committee and the University of Ghana Medical School.

### Study measures

The primary outcome of interest in this study was immune recovery status. A patient was defined as having achieved immune recovery if they reached and maintained a target CD4+ T-lymphocyte percentage of 25% following the initiation of HAART [22, 23]. CD4+ T-lymphocyte count and percentage were collected before the initiation of HAART. Pre-HAART CD4+ T-lymphocyte count and percentage were quantified after surface staining (CD3+ CD4+) by standard flow cytometry using a FACSCount system (Becton-Dickinson, Franklin Lakes, NJ) at Korle-Bu Teaching Hospital. Other concomitant demographic and HIV disease characteristics of the study participants included sex, age at study entry, age at HIV diagnosis, CDC-defined clinical category of HIV infection, and presence of TB co-infection.

### Statistical analysis

Descriptive measures (such as frequency, percent, median and IQR) were used to summarize data. Confidence intervals for immune recovery rates were estimated using methods for exact binomial confidence intervals. Fisher’s exact test was used to compare immune recovery rates between HIV patients with and without TB co-infection. Wilcoxon rank sum test was used to compare pre-HAART CD4+ T-lymphocyte count and percentage between HIV patients with and without TB co-infection. The Kaplan-Meier estimator was used to compare time-to-immune recovery between patients with and without TB co-infection, and significance was tested using the log-rank test statistic. Lehmann family of Receiver Operating Characteristic (ROC) curves were used to assess the diagnostic performance of pre-treatment CD4+ T-lymphocyte count and percentage as biomarkers for immune recovery. In this model, the ROC curve can be modeled as *y*(*x*) = *x*^θ^ where *x* represents the false positive rate, *y* represents the true positive rate, and 0 ≤ θ ≤ 1 is a measure of marker performance with values closer to zero indicating increased diagnostic accuracy [24].

## Results

The study included a total of 305 HIV-infected children who initiated HAART regimens between 2004 and 2009 at Korle-Bu Teaching Hospital in Accra, Ghana. The children were followed during the study period until they achieved immune recovery or were censored on the last day of contact. The median follow-up time was 52 (IQR = 25–81) weeks and the majority of study participants achieved immune recovery (*n* = 180, 59%) during the study period. However, approximately 24% (*n* = 72) failed to achieve immune recovery, and 17% (*n* = 53) had an unknown immune recovery status. Fifty percent (*n* = 153) of the children were males, and the median age of the participants was 5.6 (IQR = 2.41–8.02) years. Fifty-one percent of participants (*n* = 155) were TB-positive at study entry.

**Fig. 1:**
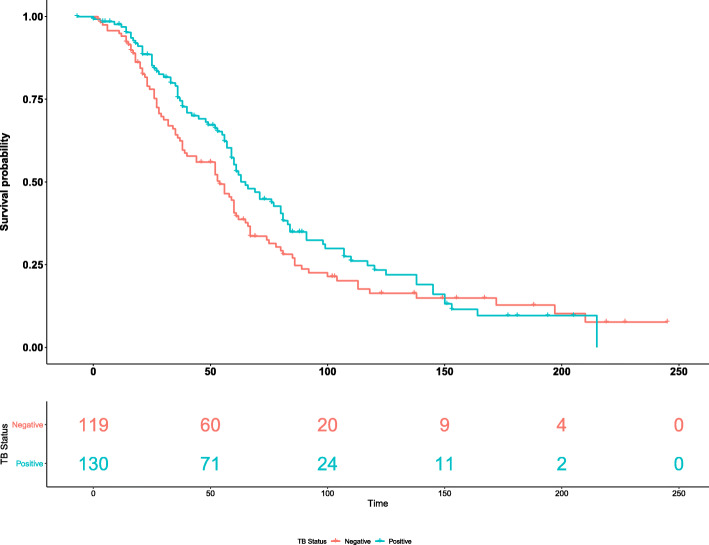
Kaplan-Meier estimates of time until immune recovery for HIV-infected patients with and without TB co-infection

To assess the effect of TB on the efficacy of HIV treatment in the study participants, we compared immune recovery rates and time-to-immune recovery between patients with and without TB co-infection. The recovery rate in TB-negative patients was 76%, with a 95% exact binomial confidence interval of 69 to 84%. Moreover, the recovery rate in TB-positive patients was 67%, with a 95% exact binomial confidence interval of 58 to 75%. The recovery rates between the two groups were not statistically significant (*p* = 0.881). Figure 1 shows Kaplan-Meier curves comparing the time-to-immune recovery between patients with and without TB co-infection. The Kaplan-Meier curves show that children without TB co-infection had shorter time-to-immune recovery at earlier time points (i.e., < 150 weeks). Median time-to-immune recovery was 65 (95% CI: 59–81) weeks for TB co-infected patients and 54 (95% CI: 40–62) weeks for patients without TB. The log-rank tests comparing the time-to-immune recovery between the two TB groups indicate no significant difference (*p* = 0.10).

**Fig. 2:**
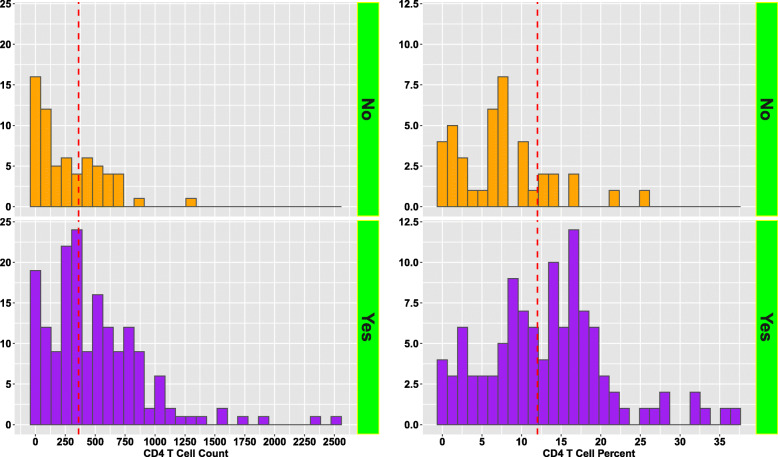
Distribution of pre-HAART CD4+ T-lymphocyte counts and percentages by post-HAART immune recovery status. The lower and upper panels of the figure are for patients who achieved and failed to achieve post-HAART immune recovery, respectively

**Fig. 3:**
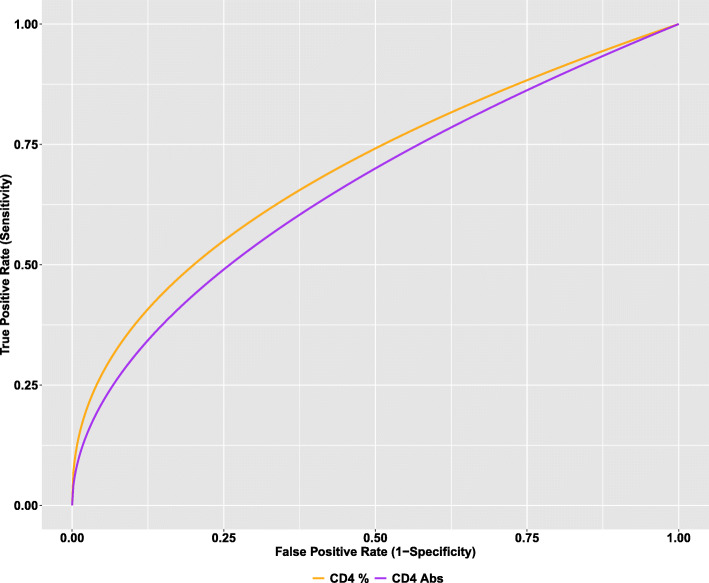
ROC curves for pre-HAART CD4+ T-lymphocyte counts and percentages

The main objective of our secondary data analyses was to assess the diagnostic performance of pre-HAART CD4+ T-lymphocyte counts and percentages as biomarkers of post-HAART immune recovery. We then compared the pre-HAART CD4+ T-lymphocyte counts and percentages between patients who achieved and those who didn’t achieve immune recovery. Figure 2 displays the distribution of pre-HAART CD4+ T-lymphocyte counts (on the left) and percentages (on the right) by recovery status. The dotted lines in the figure indicate median pre-HAART CD4+ T-lymphocyte counts (median = 361 cells/mm^3^; IQR = 129–664) and percentages (median = 12; IQR = 6.85–16.5) among all patients. As can be seen from Fig. 2, pre-HAART CD4+ T-lymphocyte counts were significantly higher in patients who achieved immune recovery (*p* < 0.0001). Median pre-HAART CD4+ T-lymphocyte counts was 391 cells/mm^3^ (IQR = 229–697) and 197 cells/mm^3^ (IQR = 48–431) in patients who achieved and did not achieve recovery, respectively. Pre-HAART CD4+ T-lymphocyte percentages were also significantly higher in patients who achieved immune recovery (*p* < 0.0001). Median pre-HAART CD4+ T-lymphocyte percentage was 13.6 (IQR = 8.50–17.6) and 7.2 (IQR = 3.1–10) in patients who achieved and did not achieve immune recovery, respectively. Figure 3 displays the ROC curves for pre-HAART CD4+ T-lymphocyte counts and percentages. Pre-HAART CD4+ T-lymphocyte counts and percentages had a 66% (AUC = 0.66; 95% CI: 0.59–0.73) and a 70% (AUC = 0.70; 95% CI: 0.62–0.78) probability of correctly distinguishing a recovered from non-recovered patient. Though the overall diagnostic accuracy of the pre-HAART CD4+ T-lymphocyte percentages was higher, there was no statistically significant difference between the ROC curves of the pre-HAART CD4+ T-lymphocyte counts and percentages (*p* = 0.754).

**Fig. 4:**
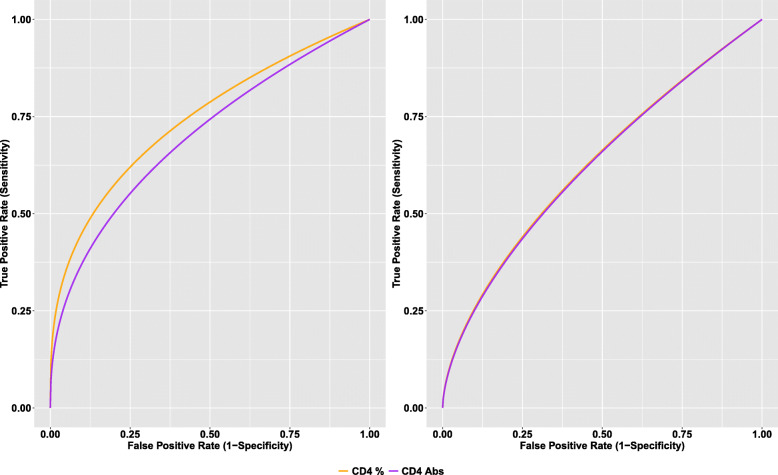
ROC curves for pre-HAART CD4+ T-lymphocyte counts and percentages by TB status. The left and right panels of the figure are for TB-negative and TB-positive patients, respectively

We then examined if the performance pre-HAART CD4+ T-lymphocyte counts and percentages as biomarkers of immune recovery varied by TB status. Figure 4 shows the ROC curve for pre-HAART CD4+ T-lymphocyte counts and percentages in TB-negative and TB-positive patients. There was no difference in the performance of pre-HAART CD4+ T-lymphocyte counts (*p* = 0.264) and percentages (*p* = 0.150) as biomarkers of immune-recovery between the two TB groups. The areas under the ROC curve of the pre-HAART CD4+ T-lymphocyte counts were 0.70 (95% CI: 0.61–0.79) and 0.63 (95% CI: 0.53–0.72) in TB-negative and positive patients, respectively. Similarly, the areas under the ROC curve of the pre-HAART CD4+ T-lymphocyte percentage were 0.74 (95% CI: 0.64–0.85) and 0.63 (95% CI: 0.51–0.75) in TB-negative and positive, respectively. Finally, due to the known variation of T-lymphocyte counts with age [25], we assessed if age of the patients impacts the performance of the two biomarkers. We found that there was no difference in the performance of pre-HAART CD4+ T-lymphocyte counts (*p* = 0.935) and percentages (*p* = 0.716) as biomarkers of immune-recovery between younger (≤5 years) and older patients (5+ years).

## Discussion

The main objective of this secondary data analysis was to assess whether the pre-HAART CD4+ T-lymphocyte counts and percentages can be used as biomarkers of post-HAART immune recovery in HIV-infected children with and without TB co-infection. The cohort we analyzed was comprised of 305 HIV infected children (age 0–13 years) who received HAART treatment between 2004 and 2009 at Korle-Bu Teaching Hospital in Accra, Ghana. Fifty-one percent of the children presented with TB co-infection. In sub-Saharan Africa, the challenges associated with monitoring pediatric HIV progression are compounded by the high prevalence of opportunistic infections in HIV-positive children, the deadliest of which is TB [20]. TB co-infection is endemic in sub-Saharan Africa and known to complicate HIV treatment regimens and increase the risk of HIV progression and death [26]. Therefore, clinical models of immune recovery based on patient status prior to the initiation of treatment are vitally important for predicting patient outcomes in resource-limited settings. Accordingly, we assessed the use of pre-HAART CD4+ T-lymphocyte count and percentage as biomarkers of immune recovery in HIV-infected children on HAART regimens and used statistical methods to adjust for important covariates such as TB co-infection.

The post-HAART immune recovery rate of patients without TB co-infection was 6% greater than that of patients with TB co-infection (76% versus 69%). However, this difference was not statistically (*p* = 0.881), corroborating previous findings [22]. Kaplan-Meier analysis of time-to-immune recovery following the initiation of HAART regimens demonstrates that patients without TB co-infection recover 11 weeks earlier on average than TB co-infected patients. However, this difference was also statistically insignificant, corroborating previous findings [22]. Median time-to-immune recovery was 65 (95% CI: 59–81) weeks for TB co-infected patients and 54 (95% CI: 40–62) weeks for patients without TB. These findings suggest that TB co-infection negatively impacts the efficacy of HAART treatment; however, the effects observed in this cohort are not very pronounced.

Previous pediatric studies have found that lower pre-treatment CD4+ T-lymphocyte count is associated with weaker immune recovery following HAART initiation [27, 28]. Results from the cohort analyzed in our study corroborate this finding, as patients who achieved post-HAART immune recovery had significantly higher pre-HAART CD4+ T-lymphocyte counts and percentages. However, our findings suggest that pre-treatment CD4+ T-lymphocyte count is not a particularly strong predictor of immune recovery. We found that pre-HAART CD4+ T-lymphocyte counts and percentages had a 66 and 70% probability of correctly distinguishing a recovered from the non-recovered patient (AUC: 70% versus 66%). However, these differences were not statistically significant. Additionally, while CD4+ T-lymphocyte percentage is preferred over CD4+ T-lymphocyte count due to its reduced variability in pediatric patients [25], we found the diagnostic performance of both markers to be comparable in the absence of covariate adjustments.

We found the diagnostic accuracy of both markers was not significantly impacted by TB co-infection status (Fig. 3). While the diagnostic accuracy of both markers was comparable within TB-positive and TB-negative subgroups, pre-HAART CD4+ T-lymphocyte count and percentage were more accurate predictors of immune recovery in patients without TB co-infection, with AUCs about 10% greater than patients with TB co-infection. The well-documented negative synergy between HIV and TB complicates concurrent treatment of both diseases, likely contributing to the poor diagnostic accuracy we observe in the TB-positive group. Finally, due to the known variation of T-lymphocyte counts with age, we assessed if age of the patients impacts the diagnostic performance of the two biomarkers. We found the diagnostic accuracy of both markers is not significantly impacted by age, confirming findings from a cohort of HIV-infected children from the USA [29]. Even in children less than 5 years of age, our results demonstrate that the diagnostic accuracy of pre-treatment CD4+ T-lymphocyte count and percentage are comparable (AUC: 67.5% versus 64.6%). While previous literature has shown CD4+ T-lymphocyte percentage to be a more clinically interpretable indicator of disease progression in young children than other common markers including viral load and CD4+ T-lymphocyte count [30, 31], these results, taken with previous findings, suggest that pre-treatment CD4+ T-lymphocyte count is an adequate surrogate marker of immune recovery in this instance. This is beneficial to the current standard of care because procedures to enumerate CD4+ T-lymphocyte count require less technical equipment and are often more feasible in resource-limited settings [32].

Previous studies have explored the predictors of immune recovery in the global HIV-infected population [33–35]. However, fewer studies have focused on children in resource-limited settings [21], and to the best of our knowledge, none have jointly considered the impact of TB co-infection. Our findings suggest that TB co-infection negatively impacts the efficacy of HAART treatment and diminishes the ability to predict immune recovery in pediatric patients. However, in the absence of TB co-infection, we demonstrate that widely available pre-treatment markers such as CD4+ T-lymphocyte count and percentage are strong predictors of immune recovery, offering diagnostic accuracy as high as 74%. These findings contribute novel models of disease progression in the context of HIV-TB co-infection.

## Conclusion

In this study, we assess the ability of pre-HAART CD4+ T-lymphocyte counts and percentages to be used as biomarkers of post-HAART immune recovery in HIV-infected children with and without TB co-infection. We find that both biomarkers are adequate indicators of pediatric immune recovery, although their diagnostic accuracy is lowered in patients with TB co-infection. Additionally, we demonstrate that median recovery times following the administration of HAART is shorter in patients without TB co-infection. These novel findings are particularly relevant to pediatric clinics in low-resource settings where HIV-TB co-infection is highly prevalent and patient monitoring is difficult. Taken together, these findings demonstrate the necessity of detection and treatment of TB in HIV-infected pediatric patients.

## Data Availability

The dataset used in the manuscript is available from the corresponding author on reasonable request.

## Abbreviations

HIV: Human immunodeficiency virus
TB: Tuberculosis
HAART: Highly active antiretroviral therapy
ROC curve: Receiver operator characteristic curve
AUC: Area under the ROC curve
CYP: Cytochrome P450
MDR: Multiple drug-resistant
IQR: Interquartile range
CI: Confidence interval

## References

[1] Joint United Nations Programme on HIV/AIDS. Global AIDS monitoring 2019: indicators for monitoring the 2016 political declaration on ending AIDS. 2018.

[2] Health TNI Of. Clinical and Laboratory Monitoring of Pediatric HIV Infection https://aidsinfo.nih.gov/guidelines/brief-html/2/pediatric-arv/59/clinical-and-laboratory-monitoring-of-pediatric-hiv-infection. Accessed 5 June 2020.

[3] Joint United Nations Programme on HIV/AIDS. UNAIDS Data 2019. 2019. https://www.unaids.org/sites/default/files/media_asset/2019-UNAIDS-data_en.pdf. 12349391

[4] Zachariah R, Reid SD, Chaillet P, Massaquoi M, Schouten EJ, Harries AD (2011). Viewpoint: why do we need a point-of-care CD4 test for low-income countries?: why do we need a point-of-care CD4 test for low-income countries?. Tropical Med Int Health.

[5] Malkin R, Keane A (2010). Evidence-based approach to the maintenance of laboratory and medical equipment in resource-poor settings. Med Biol Eng Comput.

[6] Nkengasong JN, Adje-Toure C, Weidle PJ (2004). HIV antiretroviral drug resistance in Africa. AIDS Rev.

[7] Gona P, Van Dyke RB, Williams PL, Dankner WM, Chernoff MC, Nachman SA (2006). Incidence of opportunistic and other infections in HIV-infected children in the HAART era. JAMA..

[8] Kwan CK, Ernst JD (2011). HIV and tuberculosis: a deadly human Syndemic. Clin Microbiol Rev.

[9] Corbett EL, Watt CJ, Walker N, Maher D, Williams BG, Raviglione MC (2003). The growing burden of tuberculosis: global trends and interactions with the HIV epidemic. Arch Intern Med.

[10] Getahun H, Gunneberg C, Granich R, Nunn P (2010). HIV infection–associated tuberculosis: the epidemiology and the response. Clin Infect Dis.

[11] Mayer KH, Dukes HC (2010). Synergistic pandemics: confronting the global HIV and tuberculosis epidemics. Clin Infect Dis.

[12] Havlir DV, Getahun H, Sanne I, Nunn P (2008). Opportunities and challenges for HIV care in overlapping HIV and TB epidemics. JAMA.

[13] Korenromp EL, Scano F, Williams BG, Dye C, Nunn P (2003). Effects of human immunodeficiency virus infection on recurrence of tuberculosis after rifampin-based treatment: an analytical review. Clin Infect Dis.

[14] McDonnell AM, Dang CH (2013). Basic review of the cytochrome p450 system. J Adv Pract Oncol.

[15] Wehrli W (1983). Rifampin: mechanisms of action and resistance. Clin Infect Dis.

[16] Justesen US, Andersen AB, Klitgaard NA, Brosen K, Gerstoft J, Pedersen C (2004). Pharmacokinetic interaction between rifampin and the combination of Indinavir and low-dose ritonavir in HIV-infected patients. Clin Infect Dis.

[17] World Health Organization (2010). Multidrug and extensively drug-resistant TB (M/XDR-TB): 2010 global report on surveillance and response.

[18] Bhalla AS, Goyal A, Guleria R, Gupta AK (2015). Chest tuberculosis: radiological review and imaging recommendations. Indian J Radiol Imaging.

[19] Veedu PT, Bhalla AS, Vishnubhatla S, Kabra SK, Arora A, Singh D (2013). Pediatric vs adult pulmonary tuberculosis: a retrospective computed tomography study. World J Clin Pediatr.

[20] Venturini E, Turkova A, Chiappini E, Galli L, de Martino M, Thorne C (2014). Tuberculosis and HIV co-infection in children. BMC Infect Dis.

[21] Renner L, Prin M, Li F-Y, Goka B, Northrup V, Paintsil E (2011). Time to and predictors of CD4+ T-lymphocytes recovery in HIV-infected children initiating highly active antiretroviral therapy in Ghana. AIDS Res Treat.

[22] Ghebremichael M, Habtemicael S (2018). Effect of tuberculosis on immune restoration among HIV-infected patients receiving antiretroviral therapy. J Appl Stat.

[23] Stein DS, Korvick JA, Vermund SH (1992). CD4+ lymphocyte cell enumeration for prediction of clinical course of human immunodeficiency virus disease: a review. J Infect Dis.

[24] Gönen M, Heller G (2010). Lehmann family of ROC curves. Med Decis Mak.

[25] Paintsil E, Ghebremichael M, Romano S, Andiman WA (2008). Absolute CD4+ T-lymphocyte count as a surrogate marker of pediatric human immunodeficiency virus disease progression. Pediatr Infect Dis J.

[26] Dworkin MS, Adams MR, Cohn DL, Davidson AJ, Buskin S, Horwitch C (2005). Factors that complicate the treatment of tuberculosis in hiv-infected patients. J Acquir Immune Defic Syndr.

[27] European Collaborative Study (2006). CD4 cell response to antiretroviral therapy in children with vertically acquired HIV infection: is it associated with age at initiation?. J Infect Dis.

[28] Lewis J, Walker AS, Castro H, De Rossi A, Gibb DM, Giaquinto C (2012). Age and CD4 count at initiation of antiretroviral therapy in HIV-infected children: effects on long-term T-cell reconstitution. J Infect Dis.

[29] Ghebremichael M, Michael H, Tubbs J, Paintsil E (2019). Comparing the diagnostics accuracy of CD4+ T-lymphocyte count and percent as a surrogate markers of pediatric HIV disease. J Math Stat.

[30] Palumbo PE (1998). Predictive value of quantitative plasma HIV RNA and CD4+ lymphocyte count in HIV-infected infants and children. JAMA.

[31] Hughes MD, Stein DS, Gundacker HM, Valentine FT, Phair JP, Volberding PA (1994). Within-subject variation in CD4 lymphocyte count in asymptomatic human immunodeficiency virus infection: implications for patient monitoring. J Infect Dis.

[32] Choi B-S, Park Y-K, Lee J-S (2002). The CD28/HLA-DR expressions on CD4 ^+^ T but not CD8 ^+^ T cells are significant predictors for progression to AIDS. Clin Exp Immunol.

[33] Goicoechea M, Smith DM, Liu L, May S, Tenorio AR, Ignacio CC (2006). Determinants of CD4 ^+^ T cell recovery during suppressive antiretroviral therapy: Association of Immune Activation, T cell maturation markers, and cellular HIV-1 DNA. J Infect Dis.

[34] Hunt PW, Cao HL, Muzoora C, Ssewanyana I, Bennett J, Emenyonu N (2011). Impact of CD8+ T-cell activation on CD4+ T-cell recovery and mortality in HIV-infected Ugandans initiating antiretroviral therapy. AIDS.

[35] Yeo TH, Yeo TK, Wong EP, Agrawal R, Teoh SC (2016). Immune recovery uveitis in HIV patients with cytomegalovirus retinitis in the era of HAART therapy—a 5-year study from Singapore. J Ophthal Inflamm Infect.

